# Focal Left Atrial Tachycardia in a Patient with Left Ventricular Noncompaction

**DOI:** 10.1155/2013/430862

**Published:** 2013-05-12

**Authors:** Shailendra Singh, Gulam Parihar, Rohit Rao, Vishal Goyal

**Affiliations:** Division of Internal Medicine, West Penn Allegheny Health System, 320 East North Avenue, Pittsburgh, PA 15212, USA

## Abstract

Left ventricular noncompaction (LVNC) is a rare disease caused by intrauterine failure of the myocardium to compact. The major clinical manifestations of LVNC include heart failure, ventricular tachyarrhythmia, thromboembolic event, and sudden deaths. Atrial arrhythmia usually seen is atrial fibrillation. We report a rare case of focal left atrial tachycardia in an 18-year-old patient who presented for evaluation of persistent tachycardia. Transthoracic echocardiogram showed severe systolic dysfunction and evidence of noncompaction of the left ventricle. A detailed review of ECG revealed the possibility of ectopic atrial tachycardia, most likely originating from the left side. Electrophysiology study showed sustained atrial tachycardia originating on the ridge anterior to the left sided pulmonary veins. A successful radiofrequency catheter ablation was performed at this site without any complications.

## 1. Introduction

Left ventricular noncompaction (LVNC) is a rare disease, classified as primary genetic primary cardiomyopathy [1]. LVNC is caused by intrauterine failure of the myocardium to compact. LVNC is characterized by excessively prominent trabecular meshwork and deep intertrabecular recesses of the left ventricle that communicate with the left ventricular cavity [1]. The major clinical manifestations of LVNC include heart failure, ventricular tachyarrhythmia, thromboembolic event, and sudden deaths. Atrial arrhythmia usually seen is atrial fibrillation. Atrial tachycardia is rarely reported in literature with LVNC. We report a rare case of focal left atrial tachycardia treated with radiofrequency ablation in a patient with left ventricular noncompaction. 

## 2. Case

An 18-year-old male with no past medical history presented to emergency department for evaluation of persistent tachycardia. Two days prior to admission, he had an episode of atypical pleuritic left sided chest discomfort that lasted less than 5 minutes and resolved spontaneously. The patient had no significant family history of heart disease and denied any alcohol or drug abuse. Patient was asymptomatic, and exam was benign except for persistent tachycardia in 140 s to 150 s. 

**Figure 1:**
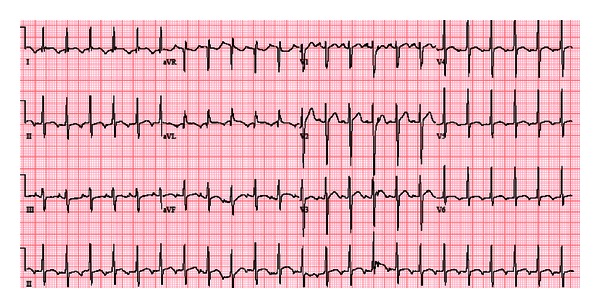
12 lead ECG on presentation. Narrow complex tachycardia with HR of 139 and nonspecific T wave abnormalities.

**Figure 2:**
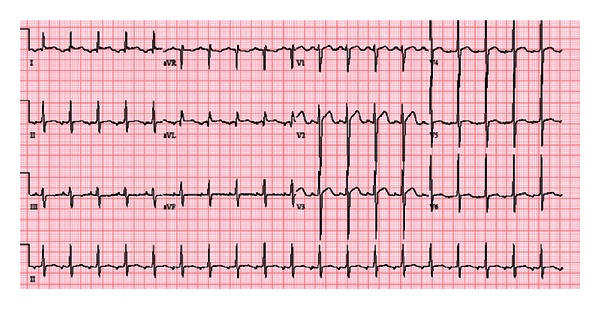
12 lead ECG. P wave become obvious as the heart rate decrease from 139 to 115. P waves are inverted in lead II and AVL, while the P waves were upright in III and AVF, suggesting the possibility of ectopic atrial tachycardia, most likely originating from the left side.

On arrival a 12 lead rest electrocardiogram (ECG) showed a narrow QRS tachycardia with P wave of 139 beats per minute (bpm) and nonspecific T wave abnormalities (Figure 1). Patient's pulse rate gradually decreased to 115, and ECG showed narrow complex tachycardia with obvious P waves (Figure 2). A detailed review of ECG revealed inverted P waves in lead II and AVL, while the P waves were upright in III and AVF (Figure 2), suggesting the possibility of ectopic atrial tachycardia, most likely originating from left side. Although his pulse rate was high, but all blood tests including complete blood count, electrolytes, thyroid stimulating hormone, D dimer and cardiac troponins, urine toxicology (only positive for cannabinoids), and chest X-ray were within normal limits. Transthoracic echocardiogram showed severe systolic dysfunction (EF: 30%) and evidence of noncompaction of the left ventricle. Cardiac magnetic resonance imaging (MRI) confirmed extensive apical and mid cavity obliteration of the papillary muscles and large trabeculations and intertrabeculation recesses in the left ventricle (Figure 4) with severely depressed ejection fraction (28%). There was no evidence of right ventricle involvement. In several views the noncompacted to compacted ratio was 2.7 to 1, easily meeting the criteria for noncompaction cardiomyopathy (Figure 4). Left atrium was moderately dilated (49 mm) with normal right atrium (38 mm), top normal left ventricle (diastolic 54 mm, systolic 62 mm). 

Electrophysiology study showed sustained atrial tachycardia originating on the ridge anterior to the left sided pulmonary veins.

**Figure 3:**
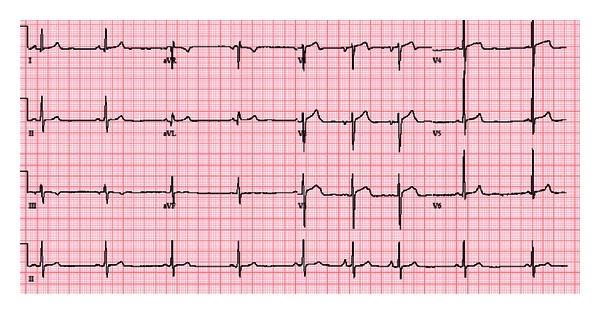
12 lead ECG after ablation of atrial tachycardia showed sinus bradycardia with sinus arrhythmia (heart rate of 53 bpm). The P waves are now positive in lead II and AVF.

**Figure 4:**
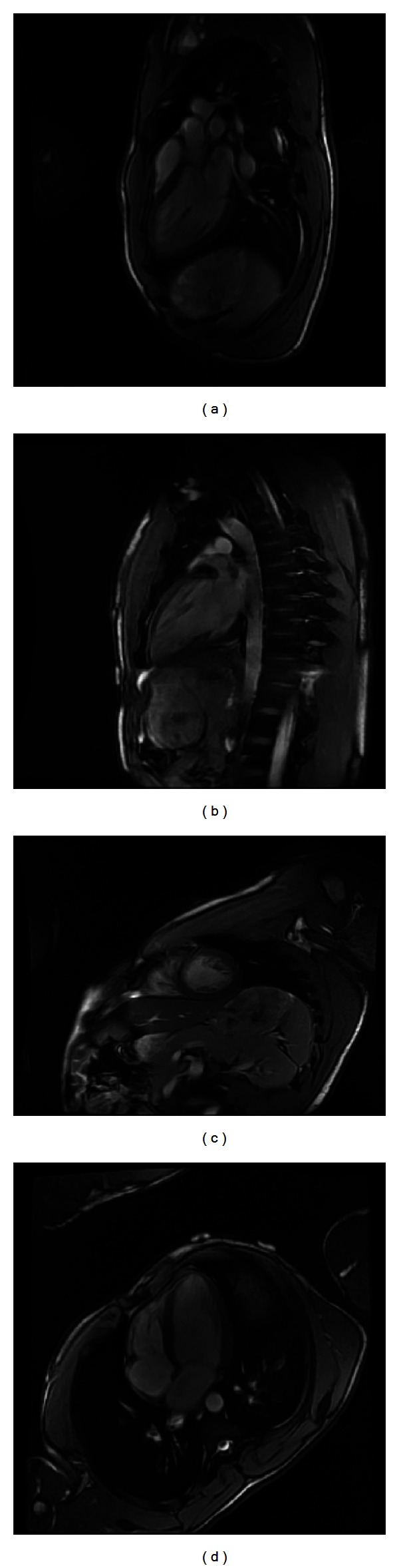
Cardiac MRI showing extensive apical and mid cavity obliteration of the papillary muscles and large trabeculations and intertrabeculation recesses in the left ventricle (a): three chamber image; (b): two chamber image; (c) short axis image; (d) four chamber image.

A successful radiofrequency catheter ablation was performed at this site without any complications. Patient was started on aspirin, beta-blockers, and ACE inhibitors and anticoagulated with Coumadin. ICD implantation was planned as outpatient for primary prevention of ventricular tachyarrhythmias. After the procedure his AT was abolished completely. Patient's heart rate improved and on 12 lead ECG done he had positive P waves in lead II and AVF (which were previously negative) (Figure 3). 

## 3. Discussion

Cardiac rhythm disturbances occur frequently in LVNC. The electrocardiographic findings are usually abnormal, but no characteristic changes are defined. The abnormalities mostly reported are branch blocks, ST-T wave changes, AV blocks, left ventricle hypertrophy, and arrhythmias [1]. Ventricular arrhythmias are the most common rhythm disturbance seen [2, 3]. The intertrabecular recesses appear to be a favorable substrate for ventricular arrhythmias. There are several case series and reports describing ventricular ectopic beats, ventricular tachycardia and/or ventricular fibrillations, and sudden cardiac deaths in these patients [1]. Atrial arrhythmia usually seen is atrial fibrillation with reported incidence of 5% [4] to 29% [3]. 

The differentiation of narrow complex tachycardias (NCT) is a commonly encountered diagnostic dilemma. In this case the patient presented with an NCT, which initially appeared to be sinus tachycardia. Sinus tachycardia generally occurs in the setting of underlying physiologic stress or pharmacologic influence, namely, fever, infection, pain, hyperthyroidism, anxiety, stimulant drug use, or hypotension, among many others. Other causes of NCTs include atrial fibrillation, atrial flutter, AV nodal reentrant tachycardia, atrioventricular reentrant tachycardia, sinus node reentrant tachycardia, unifocal atrial tachycardias, or multifocal atrial tachycardia. 

Focal atrial tachycardias, by definition, originate from a single area of the atrium (unlike MAT), the location of which governs the appearance of the P wave. Our patient had focal atrial tachycardia arising from the left side because the P waves in lead II and AVL were inverted, while the P waves were upright in III and AVF. AT usually arises following atrial surgeries or diseased atrial tissue from underlying heart disease or enhanced automaticity, if there is no underlying heart disease. We believe that LVNC with atrial involvement was likely the cause of AT in our patient.

There were no reported cases of supraventricular tachycardia in a followup of 238 patients with LVNC for 4 years with periodic Holter monitoring [5]. Although less common, SVT has been reported in patients in the setting of LVNC. Wolff Parkinson White syndrome and associated supraventricular tachycardia are described mostly in the pediatric patients with LVNC [2, 6]. Ventricular preexcitation is common in pediatric patients, and orthodromic reentrant tachycardia was reported in some of those patients [7]. One patient with AV nodal reentrant tachycardia [8] and another case of atrial flutter with extreme atrial enlargement in the setting of LVNC have been reported [9]. One case of regular sustained likely macroentry atrial tachycardia (AT) was identified [10]. However, focal atrial tachycardia is rarely described in the literature in this patient population. There is one published case of focal AT in a patient with LVNC [11], which could well represent reentrant tachycardia [10].

The patient described in this case had a focal atrial tachycardia arising from the ridge anterior to the left sided pulmonary veins, which was successfully treated with radiofrequency catheter ablation. The focal AT was probably related to the moderately enlarged left atrium. Our report suggests that although rare, involvement of left atrial myocardium is possibile in LVNC, and these patients may present with focal atrial tachycardia.
